# Allonursing in Wild and Farm Animals: Biological and Physiological Foundations and Explanatory Hypotheses

**DOI:** 10.3390/ani11113092

**Published:** 2021-10-29

**Authors:** Daniel Mota-Rojas, Míriam Marcet-Rius, Aline Freitas-de-Melo, Ramon Muns, Patricia Mora-Medina, Adriana Domínguez-Oliva, Agustín Orihuela

**Affiliations:** 1 Neurophysiology, Behavior and Animal Welfare Assessment, DPAA, Universidad Autónoma Metropolitana, Unidad Xochimilco, Mexico City 04960, Mexico; mvz.freena@gmail.com; 2 Animal Behaviour and Welfare Department, IRSEA (Research Institute in Semiochemistry and Applied Ethology), Quartier Salignan, 84400 Apt, France; m.marcet@group-irsea.com; 3 Departamento de Biociencias Veterinarias, Facultad de Veterinaria, Universidad de la República, Montevideo 11600, Uruguay; alinefreitasdemelo@hotmail.com; 4 Agri-Food and Biosciences Institute, Hillsborough, Co Down BT 26 6DR, Northern Ireland, UK; Ramon.Muns@afbini.gov.uk; 5 Facultad de Estudios Superiores Cuautitlán, Universidad Nacional Autónoma de México, Cuautitlán Izcalli 54714, Mexico; mormed2001@yahoo.com.mx; 6 Facultad de Ciencias Agropecuarias, Universidad Autónoma del Estado de Morelos, Cuernavaca 62209, Mexico

**Keywords:** animal perinatology, non-offspring nursing, fostering, mismothering, lactation

## Abstract

**Simple Summary:**

Allonursing and allosuckling are behaviors displayed by some females, characterized by nursing and feeding non-filial offspring. Although both are costly behaviors, this type of communal parenting is widespread in various species; however, not all animals display this behavior, and even among species, some differences can be observed. This review aims to analyze the biological and physiological foundations of allonursing and allosuckling in wild and farm animals. It also summarizes some current hypotheses to explain these behaviors as a strategic approach for the mother or the offspring, describing the individual and collective advantages and disadvantages and their implications on an animal.

**Abstract:**

The dams of gregarious animals must develop a close bond with their newborns to provide them with maternal care, including protection against predators, immunological transference, and nutrition. Even though lactation demands high energy expenditures, behaviors known as allonursing (the nursing of non-descendant infants) and allosuckling (suckling from any female other than the mother) have been reported in various species of wild or domestic, and terrestrial or aquatic animals. These behaviors seem to be elements of a multifactorial strategy, since reports suggest that they depend on the following: species, living conditions, social stability, and kinship relations, among other group factors. Despite their potential benefits, allonursing and allosuckling can place the health and welfare of both non-filial dams and alien offspring at risk, as it augments the probability of pathogen transmission. This review aims to analyze the biological and physiological foundations and bioenergetic costs of these behaviors, analyzing the individual and collective advantages and disadvantages for the dams’ own offspring(s) and alien neonate(s). We also include information on the animal species in which these behaviors occur and their implications on animal welfare.

## 1. Introduction

In most mammal species, attention to newborns is provided primarily by the mother [1]. Parental care entails decisions that consider the number and size of the offspring and how much energy to invest in a neonate at the cost of the reserves of parental resources for present or future offspring [2]. The mother is essential for the newborn, providing protection against predators, food, warmth, shelter, and immunological defense [3,4]. In addition, mothers aid the neonates in acquiring important information concerning their physical and social environment. During the first minutes after birth, an exchange of sensory signals occurs between the dam and their offspring(s) (sight, touch, smell, and hearing), allowing the recognition of and attachment to each other [4,5,6]. In precocial species, including most ungulates, ensuring the offspring’s survival requires establishing a mother–young bond as soon as possible after birth [7,8]. Parturient females of precocial species deliver one or more fully developed neonates that can stand and follow the mother soon after birth (around 30 min in sheep), and feeding begins as soon as this mutual recognition and attachment takes place [9]. In contrast, in altricial species, locomotor activity, sight, audition, and thermoregulation are restricted at birth [10], and the offspring depends entirely on parental care for their nutrition and growth, and, in the case of rodents, calls and ultrasonic sounds facilitate maternal bonding [11]. In these species, the newborns require constant care to nurse, feed, and provide a warm environment, representing a maternal bond formed over prolonged periods [12]. On the other hand, precocial animals (e.g., ungulates) achieve rapid inter-individual recognition, thanks to the neurochemical signaling that has a central role in the selective attachment within the first two hours after birth, to discriminate and reject any non-filial suckling after this stage [13].

The offspring of terrestrial mammals find the mother’s udder by exploring her underside from chest to teats, guided by various signals emanating from her body. Dams usually help the newborns by arching their backs and flexing their hindlimbs to facilitate access to their teats. Newborns quickly learn the physical characteristics of the udder by visual, olfactory, and thermal cues [9,14]. The neonatal behavior also depends on the type of maternal behavior. One example comes from Surti buffaloes, where neonates whose mothers were categorized as ‘highly aggressive’ or ‘attentive’ to protecting their calves reached the udder faster and fed longer than calves from females classified as ‘indifferent’ or ‘apathetic’ [15].

**Figure 1:**
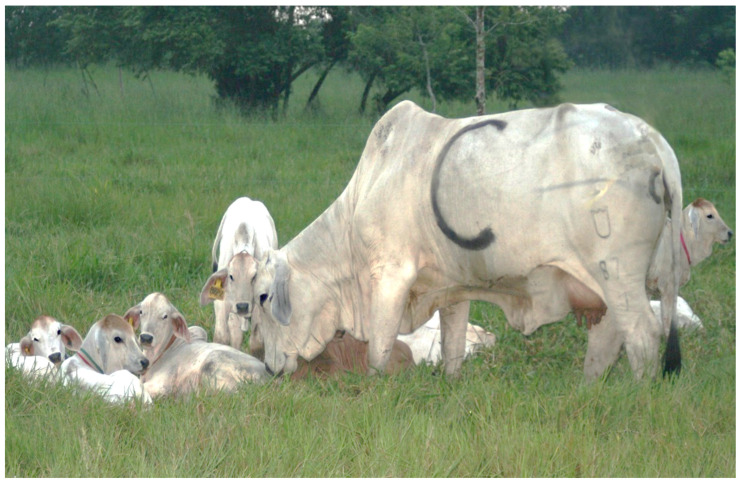
In extensive livestock production, it has recently been shown that specific cows of the herd take care of groups of zebu (*Bos indicus*) calves while their mothers graze, adepted from [22].

However, caring for the offspring is not an activity that is exclusive to the biological parents [16]. Among humans and non-human animals, there are several practices in which members of the same nest or brood care for non-filial newborns of conspecifics, either sharing care and provisioning (communal breeders), or with assistance in protection and feeding by a nonbreeding helper [17,18]. In these social systems, a pair of animals perform parent-like behaviors in non-filial young [19,20]. These include feeding, grooming, nursing, and allosuckling [20]. In perching birds, they are reported in 13% of the species and 9% in all species of birds, mammals, and fish [17]. Because they entail consequences for the fitness of animals, these behaviors can be observed in populations with stable or unfavorable conditions [17]. Some of the advantages of breeding communally at the social and group level are direct benefits, such as efficient foraging and the cooperative detection of predators [20], while the benefits towards the mother and the newborn include better reproductive performance and inclusive fitness of the mothers, weight gain, and a higher survival rate in non-filial newborns, as well as thermoregulation in the critical stage of birth [21]. Recently, Orihuela et al. [22] found some evidence of alloparental care in zebu cattle (*Bos indicus*) raised under extensive conditions (Figure 1). Similarly, Pérez-Torres et al. [23] found that zebu cows allonurse and protect non-filial calves in the first 120 days postpartum, regardless of the animal’s temperament. However, some behavioral components of protective behavior might be related with other reproductive variables, as Enríquez et al. [24] found an inverse association between the number of cows reacting to more calves and the presence of follicles, and cows displaying a more intense reaction towards their calf and estrous display.

In gregarious animals, the establishment of the mother–young bond allows the newborn(s) to be cared for by the dam, and to establish the nursing–suckling relation [25,26]. Several studies have determined that one of the most acute senses in post-parturient females is smell (olfactory) because in species such as buffaloes and sheep, dams are especially receptive to their offspring’s odor [3,27]. The maternal responsiveness facilitates acceptance of their own young or even of alien young impregnated with her amniotic fluid. In sheep, recognition of the chemical signals emitted by the lamb occurs within the first four hours postpartum, approximately, based on the dam’s detection of the specific olfactory signals emitted by her newborn [28]. Those signals are mainly produced by the wool around the anal region, the area that the dam licks most often, and where she obtains the largest amounts of chemical substances [28,29]. During the mother–young interaction, at the cerebral level, plasticity occurs in specific areas of the mother’s brain, such as the principal olfactory bulb [30].

In most mammals, the development of the maternal bond requires recognition of the offspring through olfactory, visual, and hormonal signals that culminate in said behavior [12]. Despite the need for such specific bonding mechanisms, during alloparental care, it is hypothesized that, somehow, species ignore the cost of raising a non-filial offspring and lean towards the benefits, in terms of lactation, milk synthesis, and the nutrient density of milk, to improve the development of the newborn [31].

The vomeronasal organ (VNO) is the main structure involved in maternal behavior during the olfactory recognition of the offspring, although it participates in sexual, social, and aggressive behaviors [32,33,34,35]. After birth, the body coat of the newborn is covered by the amniotic fluid [36,37], and pheromones are present in the anal region of the young [38,39]. These factors are detected by the VNO and integrated into superior and cortical structures of the brain to promote maternal bonding [40,41,42], social interactions [43,44,45,46], and sexual [47,48,49,50,51,52,53,54,55,56] or aggressive behaviors [48]. The VNO consists of sensory olfactory epithelial cells that communicate the oral and nasal cavities through the incisive duct in the roof of the nasal passage and the incisive papillae, respectively [57,58]. The neuronal axons in the VNO project dorsally to the margin of the olfactory bulb [59]. Subsequently, the signal is transmitted to the medial amygdala and hypothalamic centers to generate the aforementioned behaviors [60]. Therefore, through this olfactory system, the chemosensory mother–offspring communication is developed [61], and mammals can identify kin or conspecifics [59]. This may facilitate the development of an olfactory memory that persists, favoring attention and the survival of the offspring. In most ungulates, mothers develop exclusive care towards the young that they recognize during the first hours postpartum [62,63]. The exclusive mother–young bond allows the other to ration a valuable resource—the mother’s milk—and ensure it is consumed only by her own offspring [64], since nursing non-filial newborns would constitute a costly behavior for the female [65].

Colostrum is the first food that newborns receive, providing passive immunity, nutrition, and thermoregulation, and enhances offspring survival for several weeks or months [66,67]. The mother’s milk is the food that fosters optimal growth and development of the offspring. Interestingly, recent studies of diverse species of wild and farm animals have documented different strategies of care and attention reserved exclusively for a dam’s own offspring. One of these behaviors is denominated allonursing, which is characterized by the nursing of another female’s offspring. The term allosuckling is used to refer to the suckling of offspring from lactating females that are not the offspring’s mother [18,64,68,69,70,71,72]. These behaviors have been reported in animals such as ungulates, in swine, for example, where females deliver large litters, and in species where only one neonate is born, except for goats [73]. Allonursing is less common in monotocous (primates, cetaceans, all ungulates, except swine) than polytocous species (swine). However, in the latter, where multiple offspring are born at the same time, the energetic demand is higher than in females with a single newborn (monotocous) [74]. In the primate taxa, including prosimians (*Propithecus candidus*), allonursing has been reported in 17 of over 620 species, and some authors suggest that kin selection and nulliparity have a key role in non-filial nursing [75]. Regarding this, Dušek [76] studied the differences between both types of species and found that in monotocous animals, such as the red deer (*Cervus elaphus*), allonursing behaviors seem to benefit the offspring’s fitness, while in politocous animals (*Mus musculus*), alloparental care is performed to maximize the number of newborns [77,78]. In light of those findings, the aim of this review is to analyze the biological foundations and bioenergetic costs of allonursing and allosuckling behaviors, analyzing the individual and collective advantages and disadvantages for the dams’ own offspring and alien neonate(s), while also identifying the animal species in which they occur and their implications for the welfare of the young. In addition, some current hypotheses attempting to explain these behaviors are also reviewed.

## 2. Lactation: An Energetic Costly Period

The present work provides new findings on the costs and benefits of allonursing/allosuckling during lactation; for example, in the association between reduced maternal lactation effort and faster weaning [31], compensatory growth of the offspring, the risks of pathogen transmission or improvement of the immune response [79], and milk production increments in the cows [80], among others.

Progenitors invest in their offspring regardless of the energetical cost that could affect their own survival or reproduction [81]. This is related to the principle of “assignation”, which states that organisms have finite energy and nutrient resources to maintain all their body functions, especially growth, maintenance, and reproduction. Because every activity that an animal performs consumes energy, the process of sustaining life entails costs, since circulation, respiration, excretion, and muscular contraction never cease, not even during absolute repose [82].

An animal’s energy requirements depend on intrinsic factors, such as its basal metabolism, activity, physiological state, age, and sex [83]. Environmental features also influence animals’ energy requirements, such as the environmental temperature, humidity, precipitation, shelter, and protection from severe weather. Furthermore, the amount and quality of food available, and water consumption, affect feed intake and the possibility of animals meeting their energy requirements. Lactation is related to an animal’s reproductive success, being a process that demands particularly high energy expenditure and constitutes the most energetic aspect of mammalian biology [84]. The nutritional requirements of lactating females increase, and they maximize their food ingestion to provide the energy for satisfying their own energy needs and those of their offspring. Females in lactation produce a nutritious liquid that is well adapted to promote the growth and development of their young. The components of the energy expenditure of lactating females include the resting metabolic rate and milk energy output, both of which increase markedly during lactation [85]. This means that energy ingestion must also increase to compensate for the energy spent [86]. Marotta and Lagreca [87] observed that the energy requirements of lactating sows raised in fields correspond to the sum of the maintenance needs and milk production plus the effect of climate and physical activity. They calculated the energy required for maintenance as 110 kcal of metabolizable energy/kg^0.75^/day. Additionally, the amount of energy required for milk production during the growth of the young varied according to external environmental factors, such as confinement (19.0) Mcal/digestible energy (DE/day) vs. open-air conditions (20.7 Mcal/DE/day for autumn–winter vs. 19.8 Mcal/DE/day for spring–summer). In polygynous mammals, the energy expenditure during lactation also varies with sex, with males being more energetically demanding than females [88,89]. Furthermore, Moen [90] found that the female white-tailed deer’s largest energy outlay occurred while lactating to nurse two fawns. The lowest cost (1.5 times her basal metabolism) occurred during the reproductive period in winter.

Despite evidence that supports this asseveration, the gestation and lactation expenditure of the mother does not always follow the same pattern, since lactation is commonly consistent with intervals of food security [91]. In fact, after controlling for individual variation in a study of wild deer (*Cervus elaphus* L., an ungulate), Clutton-Brock et al. [91] demonstrated that the cost of lactation demand is higher than that for reproduction or survival.

**Figure 2:**
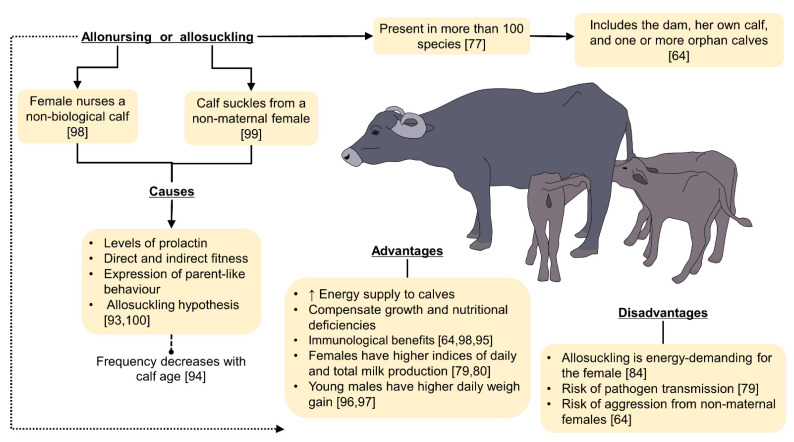
Allonursing and allosuckling causes, disadvantages and advantages to the mother and the offspring. While allonursing and allosuckling respond to endocrine and social contexts, they involve difficulties to the mother, such as the high energy demand, and to the young because of the risk of aggression of the foster mother, and the risk of pathogen transmission to the calves. However, some benefits include the compensation of nutritional deficiencies in the calves, and the acquisition of a wider range of immunoglobulins to enhance the immune system, as well as an enhancement in productivity parameters, such as daily weight gain.

Nursing can also generate conditions of physiological stress, reflected in weight loss, despite greater food consumption, which could lead to susceptibility to parasitic action, reduce fertility indices, and increase mortality rates compared to non-lactating females [92]. Considering these costs, several questions have been raised regarding allonursing or allosuckling behaviors, which are erratic in some species; for example, in wild mammals, allonursing may increase maternal mortality due to the physiological and energetic cost that lactation requires to feed not only their offspring, but a non-biological individual [91]. On the contrary, in species such as chimpanzees (*Pan troglodytes schweinfurthii*), Bădescu et al. [31] have reported that cooperative breeding involves some benefits to alloparents, such as an improvement in their reproduction, and direct and indirect fitness. This also correlates to the growth, weight gain, and early weaning of the offspring. Some benefits, costs, and causes are included in Figure 2 [64,77,79,80,84,93,94,95,96,97,98,99,100]. However, there have been reports in bat roosts, animal groups that practice communal reproduction—such as lions—and in species such as seals that do not develop strong mother–infant bonds (Figure 2). Is this a strategy that females adopt to reduce energy expenditures by not nursing their own neonates? Is it an option that newborns seek when their nutritional needs are unsatisfied? Or is it a group strategy that distributes energy outlays to ensure greater success in neonate survival?

Some stressors might come at a cost in lactation; for example, high ambient temperatures can decrease food consumption, milk production, reproductive performance, and growth of the young in farm animals [101]. In pigs, Black et al. [102] showed that when the environmental temperature increased from 18 to 28 °C, milk production decreased by 25%, consumption by 40%, and oxygen uptake decreased from 523 to 411 mL/min. Animals might succumb to hyperthermia if they cannot maintain thermoneutrality, affecting not only the energy balance, but also water, Na, K and Cl metabolism, which are important constituents of sweat, the most important thermoregulatory mechanism used to dissipate excess body heat [103]. In addition, it is possible that even some stressful situations during pregnancy might influence postpartum milk production [104] and the adaptability of the offspring [105].

## 3. The Neurophysiology of Suckling

**Figure 3:**
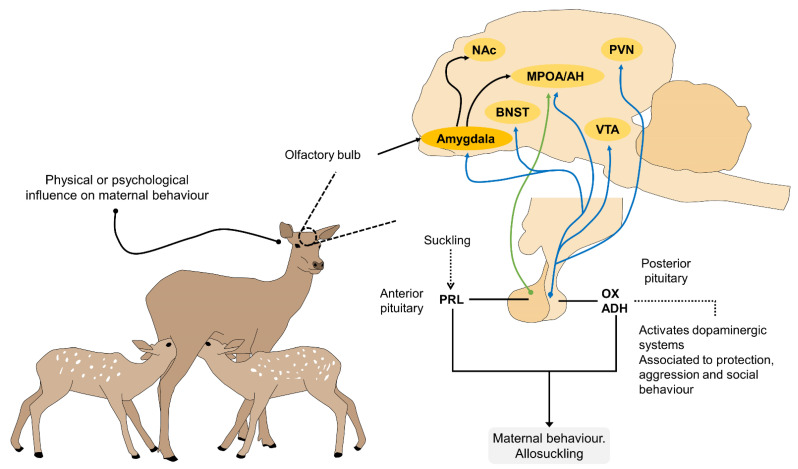
The neurophysiology of maternal behavior, milk let down, suckling and allosuckling. The main hormones associated with these processes (PRL, OX and ADH) act on different cerebral structures. The OX response is mediated by the VTA, PVN, amygdala, BNST and MPOA/AH. PRL site of action is in the MPOA/AH. The amygdala, NAc and BDNST also have a role in the reward system and maternal memory, and the former in the olfactory recognition of the offspring. ADH: antidiuretic hormone; BNST: bed nucleus of the stria terminalis; MPOA/AH: medial preoptic area/anterior hypothalamus; NAc: nucleus accumbens; OX: oxytocin; PRL: prolactin; PVN: paraventricular nucleus; VTA: ventral tegmental area, adepted from [110,111,112,113,114].

The mother’s milk is produced in the mammary gland, by extracting several chemical compounds from the blood. At the histological level, the alveolae produce the milk that accumulates and is stored in the excretory ducts and lactiferous sinuses before nursing begins. In terms of neurophysiology, the neurohormonal stimuli generated by palpation and suction of the teat—or any other stimulus that a dam associates with milking—are governed by the somatic nerves of the central nervous system that pass through the spinal cord [106]. These signals reach the hypothalamus, which releases oxytocin, principally, though a whole cascade of hormones is involved, including vasopressin from the posterior lobe of the hypophysis [107,108], which travels through the bloodstream to the mammary gland. Oxytocin is directly responsible for the myoepithelial contraction that releases the milk from the lactiferous ducts into the cistern of the gland and, from there, to the teat or nipple where it is ingested by the infant [109]. The neurophysiology of suckling or allosuckling is summarized in Figure 3.

The mechanism of suction, or suckling, has been associated with inhibition of the releasing of the luteinizing hormone (LH) and the return to ovarian cyclicity after birth. In beef cows, the episodic secretion of the gonadotropin-releasing hormone (GnRH) by the hypothalamus modulates the hypophyseal pulsatile release of LH. Although the hypothalamic content of GnRH is not affected by the condition of lactation, the concentrations of GnRH in the pituitary portal system are suppressed by the act of suckling. During the postpartum period, the patterns of LH secretion remain at sub-optimal levels during the development of the preovulatory follicles. The renewal of cyclicity and ovulation is delayed until the frequency of the LH pulses increases to the threshold observed during proestrus. Once the offspring is removed, there is a delay of 24–48 h for the frequency of the LH pulse to increase, but inhibition is often reestablished quickly after the return of the calf in the early puerperium period [115]. Silveira et al. [115] explored the possible role of maternal behavior in anovulation mediated by lactation, hypothesizing that identifying the suckling calf is a critical determinant of anovulation induced by nursing. Those researchers randomly assigned 27 crossbreed meat cows to one of the following three study groups: dams that nursed an alien calf (*n* = 11); dams that nursed their own calf (*n* = 8); dams whose calves were separated for 6 days (*n* = 8). They observed that forced suckling by alien calves did not attenuate the release of LH in the cows after extraction by their own calves. In addition, the anovulatory intervals of the allonursing cows were similar to those of the weaned, non-nursing cows (study group 3). Moreover, neither the suckling posture nor the stimuli that resulted from contact with the udder affected LH secretion. The authors concluded that the maternal bond is important, but not essential, for anovulation mediated by lactation.

On the other hand, stimulation of the sow’s teat by both filial and non-filial offspring benefits milk production. In pigs, constant teat stimulation by the piglet has been shown to maximize colostrum production [116,117], enhance mammary gland growth [118], and promote better local blood circulation [119]. Therefore, although the effect of nursing frequency, littler size, and weight, among others, can affect milk production, massaging of the teat, and the consequent hormonal and circulatory changes increase milk production in females [120]. This enhanced mammary gland development has also been reported in meerkats (*Suricata suricatta*), in whom the parity (primiparous and multiparous) is associated with greater tolerance to allosuckling from alien pups [121].

## 4. The Main Hypotheses Explaining Allonursing and Allosuckling

**Figure 4:**
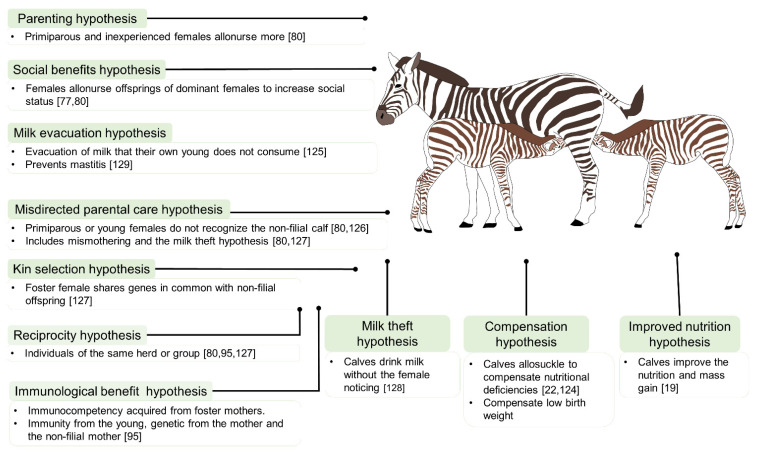
The main hypotheses explaining allonursing and allosuckling. Some of the hypotheses of these two cooperative maternal behaviors include genetic origins of the foster mother and the non-filial young, social and health benefits for the female, and advantages to the offspring to compensate and improve nutrition and immunocompetency.

There are some theories that attempt to explain why females accept non-filial offspring and why young allosuckle [80]. It may be intentional (i.e., when a dam is aware that the newborn she nurses is not her own, but allows it to accede to her udder anyway), or due to errors in breeding, where the dam is unaware that she is nursing an alien newborn, perhaps because she does not recognize it or fails to identify it while nursing it together with her own newborn [73]. Evolutionary theories on the origins of allonursing behaviors have proposed various phenomena, as follow: reciprocal altruism, selective parenting, poor targeting of parental care, a resource-optimizing strategy (care, food) [122], and maintaining social stability [65]. To date, the literature includes eight hypotheses regarding this kind of cooperative breeding, where the cost–benefit relation has a relevant role in the decision [92]. These include genetic bases as well as social and immunological benefits for both the offspring and the foster mother [123]. In addition, it is important to emphasize that all hypotheses are usually related to allosuckling and allonursing, so they are not mutually exclusive [92], with the exception of the improved nutrition and the compensation hypotheses. The main allonursing and allosuckling hypotheses are summarized in Figure 4 [19,22,77,80,95,124,125,126,127,128].

### 4.1. Allonursing: A Strategy Adopted by Dams?

#### 4.1.1. Kin Selection or Selective Parenting

This hypothesis describes the relationship that is established between females and offspring that share kinship, or between those who are close relatives, where dams only allow alien offspring to suckle if they share genes of common ascendence, as this ensures that the infants will survive to disseminate those genes in the group [73,77]. Analyses of the association between allonursing and litter or group size suggest that this is more common in primiparous females than multiparous species [122]. Another observation is that allonursing occurs more often in reduced groupings with close kinship [122]. In monotocous species, such as African elephants (*Loxodonta africana*), the prevalence of calves attempting to feed on individuals within their families, or closely related individuals, is approximately 78.9% [129]. However, this hypothesis does not apply to all species. Banded mongoose (*Mungos mungo*) is a species where the collective care of the offspring is performed by an adult who is responsible for feeding, protecting, and transmitting skills to single young. It has been reported that allonursing in these animals does not follow a genetic distinction between young. However, the durations of care and interaction are longer for those with genetic heritage, and sex-specific behavior has been observed in mongooses, where females care more for same-sex, non-filial newborns [130]. In water buffaloes, this kinship relationship did not show influence in a herd of 30 buffaloes. Of 570 allosuckling events, 351 were from alien calves; moreover, the calves of sisters and half-sisters had a higher success rate when requesting allosuckling from genetically unrelated mothers [90]. In otariids, such as *Arctocephalus australis* that practice philopatry shared breeding areas, allonursing has been observed to improve reproductive success [92].

In animals in which dams group according to kinship, alloparental care does not have an association with a greater variation in kinship, but is more frequent among species with litters [78]. In this case, the relative inversion by descendance is likely reduced, together with the additional costs of lactation, by dividing them among various newborns. The findings reported by MacLeod and Lukas [78] suggest that feeding alien offspring can progress rapidly when the additional costs for all dams are reduced in relation to the benefits it represents for their offspring. However, this distinction that some animals can make about kin selection among offspring has also been related to a high risk of aggression by unrelated herd members [131]. While the kin selection hypothesis can be prevalent in rodents, in the Sinai spiny mice (*Acomys dimidiatus*), allonursing was attributed to the maternal experience of the animals and to misdirected parental care [132]. Similarly, in the domestic reindeer (*Rangifer tarandus*), Engelhardt et al. [133] found no support for this hypothesis in 25 pairs of animals (mothers and calves). From 5176 successful allosuckling events, no correlation was found between the relatedness of the reindeers and the acceptance of non-filial calves.

The inclusive fitness/kin selection theory cannot explain how helping behavior between non-kin, and direct and indirect fitness can interact [134,135]. Alloparents are not always related to the offspring they help [136,137]. The direct fitness of helping is likely also important between kin [135]. Variation in relatedness explains approximately 10% of the variation in provisioning behavior in avian and mammalian cooperative breeding social systems [138,139]. The importance of indirect fitness is overestimated, whereas the importance of direct fitness is underestimated [140].

#### 4.1.2. Reciprocity Hypothesis

Reciprocity is observed when females allonurse the non-filial young of mothers who have previously fed their offspring, which some authors considered to be some kind of reciprocal altruism, where the animal nurses or helps other individuals at the expense of their wellbeing [141]. This relation is proposed when free-roaming or captive dams spend substantial amounts of time separated from their offspring, though this does not seem to be an essential pre-condition for allonursing, since it occurs in otariids when the mother is absent, feeding in the sea, while her newborn is being fed on land by an alien female. Under those circumstances, mothers cannot be sure which female is reciprocating [92].

Tendencies in reciprocity imply a benefit of group rearing, since it promotes an improvement in the fitness of both females who assist in any milk deficiency of the biological female, and contributes to reinforcing the calf’s immunity, which are aspects that involve other hypotheses that will be addressed later [80]. In a study with reindeers (*Rangifer tarandus*), by Engelhardt et al. [127], with 25 does, it was reported that females allonurse by reciprocity among lactating females from the group. Lactating wild sows in social groups also tend to accept suckling from alien piglets. This behavior is considered as inclusive fitness, where the piglet benefits from the alloparental care and the sow can forage without exposing its offspring to environmental and predator dangers [131]. Similarly, in wild Yellowstone bison (*Bison bison*), Jones and Treanor [142] observed cooperative behaviors, such as cleaning of the newborns, and even mutual consumption of the placentas, which are activities that could be a maternal temporary relief and increase the chances of the young’s survival. In contrast, in water buffaloes, the reciprocity hypothesis has not been linked to allomaternal care [79].

There are other types of reciprocity, including generalized and indirect. In the first type, there is an effect that affects the entire population and increases the probability that all individuals will cooperate after social interaction, regardless of the recipient. In contrast, individuals practicing indirect reciprocity decide to help and cooperate with others despite whether they have received help from them or not [143]. The frequency of interaction is important in this type of reciprocity and involves a certain degree of evolutionary cooperation in the population.

#### 4.1.3. Parenting Hypothesis

Among some terrestrial species, nursing the young of other dams in a proportion similar to their own newborns—collective lactation—may allow mothers to improve their reproductive performance compared to dams that do not share their milk with other offspring in the group [73], particularly in primiparous females [80,92]. An example of this is observed in female water buffalo, in which 97% of 30 dams allonursed alien offspring due to the mother’s experience [90]. Additionally, studies show that dams who feed their own calves and non-filial ones have an increased quantity of produced milk [79,80]. Furthermore, studies have determined that dams nurse alien offspring to improve their maternal abilities. This may help explain why inexperienced dams, or females that have yet to reproduce, perform allonursing behavior more often than experienced ones, to learn to parent [73]. Spontaneous allonursing events were reported in dwarf mongooses (*Helogale parvula*) in the work of Creel et al. [144]. In this study, females with spontaneous lactation could nurse and suckle non-filial young. In the case of otariids, however, juvenile or inexperienced dams have not been observed to participate in allonursing, perhaps because they only give birth once a year, so it would be difficult for them to obtain maternal experience by nursing or caring for two offspring at the same time [92].

This theory also includes non-breeding females or those with pseudopregnancy and spontaneous lactation, such as carnivores, particularly canids, in whom alloparental care has also been reported [145]. Additionally, in these taxa, allonursing is correlated with high concentrations of prolactin and oxytocin, and low concentrations of testosterone and glucocorticoids [145]. High concentrations of these neurochemicals have been associated with an increase in cooperative and maternal behaviors [146], as well as milk ejection. They bind to their respective receptors in the hypothalamus, the posterior pituitary gland, and other structures, such as the mammary gland [145]. When a filial or alien young suckles a female, this stimulates the receptors located in the teats and induces the secretion of prolactin and oxytocin [19]. On the other hand, testosterone induces paternal behaviors [145], while the reduction in glucocorticoids is associated with improved fitness in communally breeding species, such as rodents [147].

#### 4.1.4. Social Benefits Hypothesis

Cooperative nursing and feeding of non-filial offspring are also associated with social benefits within the group, such as a reduction in aggression by other members, a reduction in infanticide, and the maintenance of social rank [69]. Some reports describe allonursing as an extreme communal form of raising offspring in mammals, a phenomenon that may be due to a herd’s social stability, since it rarely appears in unstable groups; for example, this behavior is often observed in artiodactyls, but rarely in equines [65]. Pluhácek and Bartosová [148] reported, for the first time, allosuckling in a common hippopotamus herd (*Hippopotamus amphibius*), composed of one adult male, one male offspring, and two multiparous females, who were the mother and grandmother of the offspring. Although these animals were in captivity, this type of allosuckling event could be related to kin selection and a certain type of social benefit, where the daughters preferred to stay and rear their offspring in the same group, to protect and increase the survival rate of the young. Similarly, in species with a strong social group structure, such as meerkats (*Suricatta suricatta*), allonursing practices are common, mainly in mothers that have lost their young. These animals do not only allosuckle non-filial young, but also engage in anti-predatory activities, such as staying alert to protect the young, involving the entire community [149]. A similar situation was observed in wild cavies (*Cavia aperea*). Cavies usually live in groups of one to four adults, where the mother–offspring bond is strong. In these animals, alloparental care is unusual. However, it was observed in a group of one male, two females, and four young, in which social structure, food availability, calving time, group adaptation, and cohesiveness may be factors that contribute to the presentation of this behavior. In contrast, in reindeers, cooperative care of animals has not been shown to provide a social benefit for the mother [133].

Another benefit that is associated with social improvement includes the group augmentation hypothesis, which is considered to be a way to enhance indirect fitness [150]. This hypothesis refers to a reproductive improvement in breeders, survival of the herd, and keeping a large population of individuals within a group that can promote long-term benefits [151]. It is important to consider that the group augmentation hypothesis can also enhance direct fitness [151,152,153].

#### 4.1.5. Milk Evacuation (Milk Dumping)

The milk evacuation hypothesis is related to the excess of milk and the inflammatory consequences that this causes in the dam. This hypothesis states that allonursing helps to evacuate milk that the dam’s own infant does not consume by offering it to alien offspring. Among wild animals, this practice could have the advantage of reducing the female’s weight, improving their agility when being hunted [73]. This pattern is observed more frequently in polytocous species, due to the large litter size, where it also constitutes a benefit by reducing the cost of milk production, since they are species with large litters by nature [104]. In the evening bat (*Nycticeius humeralis*), Wilkinson [125] stated that mothers who produce large amounts of milk allonursed alien pups to prevent mastitis development and promote milk evacuation.

In some species, milk production has also been associated with allonursing. Allosuckling also increases teat stimulation, with consequent oxytocin release and increased milk production in some cases. This observation is similar to that found in a group of 35 buffaloes of the Murrah, Jaffarabadi, and Mediterranean breeds. The buffalo females that fed alien offspring showed higher levels of daily milk production and total production (peak of 6.96 ± 0.3 kg/day, and 1853.0 ± 76.2 kg, respectively), especially in those who allonursed male calves [97]. In contrast, as mentioned by Paranhos da Costa et al. [96], the daily milk production of buffaloes suckled by female calves was higher than in those suckled by males (4.56 ± 1.01 vs. 4.181 ± 0.60, respectively), although the bull-calves obtained greater weight gains (0.49 ± 0.13) compared to the heifers (0.39 ± 0.11). Something remarkable regarding this hypothesis is that sharing milk with non-filial young does not imply a nutritional imbalance for the filial calf, since the milk offered represents an amount that the filial calf cannot consume from its mother [78]. Similarly, in females who have lost their young, due to miscarriages or other events, alloparental care benefits the health of the udder by promoting the expulsion of milk and preventing mastitis [129].

#### 4.1.6. Misdirected Parental Care

In contrast to these proposals, but keeping in mind that the priority for the females of gregarious animals is to optimize the care and feeding of their own offspring, allonursing has also been conceived—negatively—as an erratic or misdirected behavior, attributed to dams who fail to perceive that they are nursing an alien newborn. This hypothesis includes the allomaternal care events in which the female nurses and feeds non-filial offspring without recognizing it as alien young, or perhaps she is aware that an alien newborn is stealing her milk, but does not reject it. Some authors infer that in this case, the benefit of group rearing is greater than the cost of being vigilant and preventing an alien calf from stealing the milk [98]. Within this hypothesis, mismothering and milk theft are also included. Both are considered to be maladaptive processes [133]; however, although some authors use both without distinction, the hypothesis of milk theft is often associated with a trait linked to the newborn rather than to the mother [154].

Zapata et al. [99] reported such apparent unawareness, or lapses in attention, among wild guanaco dams in the Parque Nacional Torres del Paine in Southern Chile. They observed two guanaco calves, about one month in age, being nursed simultaneously by a female. One was her own young, and the other an alien. Both newborns appeared to be healthy and in a good nutritional condition. During the periods of allosuckling, the dam apparently did not realize that the alien calf was positioned behind her own offspring. Even after moving her head to look behind, she showed no active signs of rejection, such as walking away, kicking, or spitting; she did not reject the alien offspring. The authors hypothesized that her behavior might be based on a cost–benefit balance, in that the milk thieving produced less pathological risk than the energy expenditure required to remain sufficiently alert to detect, recognize, and reject the alien calf [73].

In pinnipeds, such as the South American fur seal (*Arctocephalus australis*), recognition of the young, after foraging trips to the sea, is performed through vocal and olfactory signals. The pup does not usually set connections with members other than its biological mother. However, low percentages of allosuckling have been observed (3.37%), and are attributed to misdirected parental care when the mother returns from the sea and cannot recognize the pup. It can also be motivated by the hunger of the offspring, who approaches the non-filial mother to suckle, with the risk of suffering aggression from them, while the mother returns. Another likely reason could be the individual characteristics, such as behavioral syndromes and the personality of the offspring that performs the mentioned behaviors [92]. This result was similar to that reported in Steller sea lions (*Eumetopias jubatus*), species in which the period required to raise a single pup is long. Maniscalco et al. [126] observed only 28 cooperative breeding events, where eight primiparous females devoted more time to non-filial care (median of 359.1 s), due to maternal inexperience, in comparison to the same number of multiparous females (29.8 s), who also rejected any approach unless they were found sleeping. Furthermore, adoption was described in a mother who had lost her pup after a few days of birth. Although another 19 pups died, no other adoption case was reported, so the authors did not consider it as misdirected parenting, since the adoptive mother had previous maternal experience. Lastly, even though females are considered to give allomaternal care because they are not capable of identifying filial from non-filial young, selectivity in nursing certain offspring has also been reported, which can be interpreted as the mother understands that it is not her young, but the benefit of nursing the alien offspring is greater [125].

The studies mentioned above show that the maternal strategy of allonursing has significant benefits for the newborn fitness that can also strengthen the immune system. Examples of these benefits are accelerated growth, better nutrition, and survival of the newborn among the group [155]. This is due to the protection of the young by other members of the herd and the reduction in time between meals when several mothers feed non-offspring [78]. Similarly, this could be due to licking, huddling, thermoregulation of the newborn, and the improvement in their immunocompetence by the transmission of immunoglobulins and lymphocytes through the milk of different mothers [156]. For the mothers, the improved reproductive capacity of females has also been reported [156]. In addition, there are some limited benefits to the mother when parenting is directed at close relatives [78], although in house mice this is mentioned as a direct adaptive benefit [157]. These benefits, however, entail a cost for the female, since lactation requires the availability of a greater energy load [78]. Various explanations have been proposed to elucidate the benefits of this costly behavior for dams, most of which are focused on adaptive aspects derived from maternal care. One suggests that females nurse to acquire experience in maternal care (as mentioned in the misdirected parental care hypothesis), while others posit allonursing as an effective means of evacuating excess milk that can accumulate and cause pain [78], foster systemic or mammary gland infections [92], or impede mobility. Still, others propose that it increases the probability of reciprocal behaviors, or provides indirect benefits when dams nurse the offspring of females in the group who are related by kinship ties (as stated in the kinship hypothesis) [78]. In pigs, the teat stimulation of filial and non-filial offspring has been shown to increase milk production in the current or subsequent nursing and allonursing behaviors [158]. However, there are some exceptions, and additional scientific studies must be conducted to clarify the precise causes of this maternal behavior and the circumstances in which it is performed in a wider range of species [122]. Future studies must consider the non-nutritional aspects of allonursing, such as when the newborns do not consume the mother’s milk, but the mother accepts the interaction. In this situation, why are mothers giving offspring access to their udders? Understanding whether this effect is due to a benefit to the mother, in terms of milk production by stimulating receptors in the teats, or whether the mother simply responds to a biological need of the calf, could expand the current understanding of community care of the young. Similarly, the immunological benefits that can be obtained from communal breeding is another field that needs in-depth study to discern the physiological benefits of allosuckling.

### 4.2. Allosuckling: A Strategy for Offspring?

Allosuckling is often correlated with milk thieving by alien offspring and in females who allonurse after losing an infant [90]. This behavior provides advantages for newborns by allowing them to satisfy their nutritional needs by stealing milk from an alien dam [77,122].

#### 4.2.1. Milk Theft

**Figure 5:**
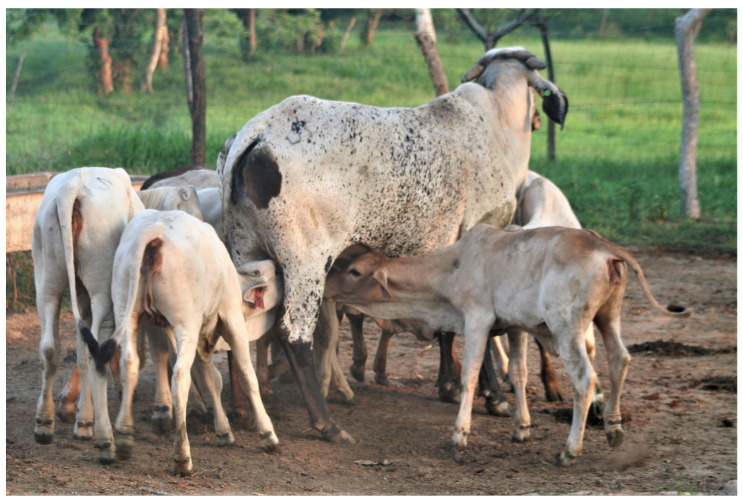
Even though, in most ungulates, cows develop exclusive care towards the young that they recognize in the first hours postpartum, adepted from [56], some cows display no rejection to calves from other cows. In practice, these cows may be selected for the rearing of orphaned calves or for those whose mothers do not produce enough milk.

Milk theft constitutes one of the most studied hypotheses, in which non-filial offspring steal milk from non-filial mothers by placing themselves in positions where the female cannot see them, or by doing so in conjunction with filial calves to increase the probability of feeding success, as in the case of giraffes [123]. If the infant’s own mother cannot satisfy its needs—because she died, is primiparous or inexperienced, or has inadequate milk production—it will turn to an alternative source and begin to steal milk from a substitute female [73]. This condition has been reported in domestic animals, such as bovines of the Curraleiro Pé breed in Brazil, where allosuckling occurred in the presence of the filial newborn and the non-filial calf. In those cases, fattening cows apparently did not discriminate between grooming and nursing alien offspring [159]. These cows, with low selectivity for their calves, have some practical uses within some cattle farms (Figure 5). In the river buffalo (*Bubalus bubalis*), allosuckling has been linked with maternal inexperience in young females, whose hungry calves may begin to steal milk from alien mothers [73] to satisfy their nutritional needs and ensure their survival. Among wild animals, reports on Iberian red deer (*Cervus elaphus hispanicus*) indicate that if the mother has low milk production, the offspring tend to suckle more from other females. Studies of this species thus propose allosuckling as a compensatory response to a reduced supply of the mother’s milk [73].

Regarding the observations of attempts by newborns to perform allosuckling, recent studies that compare the results of research from the 1990s show an increase in the percentage of time that offspring perform this behavior, over the 10% of total time that offspring devoted to suckling from alien dams in the 1990s. Attempts to explain this difference cite the controls imposed in earlier research, advances in the tools provided by the science of etiology, and increases in the frequency of animals being held in captivity, in intensive production systems that may trigger this behavior as part of a pattern to protect the offspring. The observations, in this regard, show that both domesticated and wild animals kept in captivity show increased allosuckling behavior by the offspring, reaching levels as high as 50 and 43% in river buffaloes and fallow deer (*Dama dama*), respectively, as well as in wild mouflon (*Ovis musimon*). Studies of captive Iberian deer found that milk ingestion by allosuckling reached levels as high as 37.8% of all nursing events when performed under group conditions [73]. Zapata et al. [99] analyzed 123 h of video recordings of wild guanacos, but observed only one case of allosuckling. They described the event as follows: While the mother was caring for her own calf in an antiparallel position, an alien young accessed the female in a parallel position behind the filial offspring. When the non-filial calf began to suckle, the mother smelled and recognized her own young. The dam showed a passive attitude after smelling her calf and remained still for around 30 s. The alien calf took advantage of that time to suckle, separating from the female when the group began to move. The researchers concluded that this behavior is uncommon among free-roaming guanacos. This agrees with Murphey et al. [79], who mentioned that the acceptance rate of an alien water buffalo calf increases when filial calves are feeding.

Concerning the position adopted in cases of successful allosuckling, Olléová et al. [65] found that the most common orientation of the Grevy’s zebra foals was inverse parallel, followed by the parallel position, with very few cases of the perpendicular posture. Their study documented allonursing in zebra species housed at the Dvur Králové Zoo in the Czech Republic; there were 29 foals and 23 mares of plains zebras in three herds, 8 foals and 18 mares of Grevy’s zebras (*Equus grevyi*) in one herd, and 6 foals and 9 mares of mountain zebras. They observed the following four specific events: successful nursing (uninterrupted from 5 to 60 s); attempts by foals to suckle at their mothers’ udder (<5 s or when the mother did not permit contact with the teats); attempts to allosuckle; successful allosuckling. They also noted the nursing foal’s position relative to the mare (inverse parallel, perpendicular, or parallel). Their findings for the Grevy’s zebras revealed a relation of 1729 successful filial nursing attempts vs. 824 unsuccessful attempts, with 13 successful allosuckling events vs. 117 failed attempts. Among the plains zebra foals, both successful and unsuccessful attempts at nursing from their own mothers were more frequent (4614 successes vs. 3192 failures). No successful attempts at allosuckling were observed, and only one failed attempt was recorded. The observations of the mountain zebras showed greater success in nursing behavior with the foals’ own mothers (843) compared to failed attempts (296), but only one, unsuccessful, attempt at allosuckling. The authors concluded that Grevy’s zebra mares show greater tolerance towards alien foals than those of the other two zebra species, perhaps due to their social bonds and the lack of a hierarchy in this species compared to the other two. In addition, allosuckling may occasionally occur among Grevy’s zebras, only during a specific period, as has been observed in captivity. Finally, the foal’s perpendicular position means that it is easily detectable by the alien dam, but even so, she allowed it to nurse. This led the authors to suggest that the dams’ position in the group’s social system could influence the frequency of allosuckling in this species.

In semi-domestic reindeers (*Rangifer tarandus*), milk theft and antiparallel positions were attributed to allosuckling events [127], in which females recognize non-filial offspring (misdirection does not occur) and kin selection has no relevance in their care. A similar case was observed in Bactrian camels (*Camelus bactrianus*); this theory has also been reported as the reason for allosuckling, a prevalent behavior of older calves allosuckling, motivated by the weaning of the biological mother [146]. Regarding Bactrian camels (*Camelus bactrianus*), Miková and Sovják [73] observed that animals held in captivity seemed to exhibit allosuckling behavior similar to that observed in other animals. Similarly to other ungulates in the wild, this species lives in social groups, which is a fact that seems to favor allosuckling behavior by offspring. These groups of camels are made up of mostly females with their young. During the reproductive period, they are joined by males that fight to maintain their harem. Groups maintained in captivity are similar—females and offspring, adults, and males—but males are allowed to mate with the females during the estrus period, and they remain together year-round.

There is no scientific evidence suggesting that captivity generates allosuckling in these offspring, which remain with their mother for 1–2 years, a period of virtually exclusive care, though the dams do not perform active maternal behaviors, such as cleaning the calf or bringing it close to the udder [73]. This is in contrast with other domesticated animals’ characteristic behaviors, such as buffaloes, cows, and sheep [64]. In contrast, there are reports that allosuckling in guanacos held in captivity ranges from 4.1–40% of all nursing episodes. This suggests that the indices of allosuckling in one species can vary considerably, suggesting the need to contemplate environmental conditions as well [99]. In a study of Pampas deer (*Ozotoceros bezoarticus*, Linnaeus 1758), Villagrán [160] mentions that, under conditions of semi-captivity, the duration of the mother–infant bond may be prolonged naturally, due to space limitations and the increased frequency of contact among individuals. This may also facilitate allosuckling behavior.

When alien offspring allosuckle, there are certain modifications in the signals of the filial behavior of both the mother and the young. Silveira et al. [115] studied 24 primiparous crossbreed heifers and 11 multiparous beef cows (parity 2–9) and observed that after the implementation of a regimen of controlled nursing, the dams initially smelled the alien calf, often thoroughly, but ignored it later when it attempted to nurse. When the calves born to the beef cows were exposed to the alien mother, they attempted—almost without exception—to avoid face-to-face interaction, instead of concentrating their efforts to bypass a board placed to block access to the udder. In contrast, the dams of that group generally responded to their offspring by exchanging vocalizations, smelling, licking, and staying close during periods of face-to-face interaction. Another behavior observed frequently was pseudo-suckling of the dam’s chest area and neck. The filial calves, in comparison, sought face-to-face interaction and waited, showing some patience, for the board to be removed, so they could begin to suckle. The filial calves of the cows were nursed completely and adequately shortly before the 10 min period elapsed. They were then removed easily from the corral where the mother was held. The alien calves nursed more vigorously, more often, and apparently touched the teats with greater force. Invariably, the alien calves had to be forcibly separated from the teats after 10 min and then expelled from the pen. The typical nursing posture adopted by all calves was either the classic inverse parallel position at a 45° angle, or perpendicular to the mother’s body [115].

In general, this hypothesis applies better for monotocous females, for whom milk production means a high energy demand, so they do not usually share their production voluntarily [78]; it also applies for species whose litter size is large, so it is more difficult for the mother to identify non-filial offspring [77]. Although, König [157] considers that in polytocous species, the milk theft hypothesis is less probable. In house mice, communal nursing represents a benefit for the pup, regarding thermoregulation, defense against predators, and feeding. In the African lion, the females may recognize the alien newborn, but perform allonursing nonetheless.

#### 4.2.2. Compensation

In primiparous females that usually give birth to young with low birth weights, or whose amount of milk is not enough to cover the needs of the calf [88,129], compensation by allosuckling has been reported in cattle and buffaloes [161]. Zapata et al. [124] have reported that, in guanacos, the mothers of calves that request allosuckling have a low body condition (*p* = 0.02) and nutritional deficiencies that can lead the young to feed on other dams, who accept the interaction 57% of the time. On the other hand, female water buffalo that suckle other calves tend to restrict the amount of milk they consume, so the calf prolongs the sucking time and performs allosuckling to meet their nutritional requirements [93]. It is important to note that allosuckling should not be seen as a negative aspect, or isolated from the characteristics of productive units and conditions of captivity, because it impacts the offspring’s productive performance, just as birth weight, birth order, sex, and age do [73]. Víchová and Bartoš [94] reported that female offspring suckle more from alien mothers than male calves, and in the young of fattening cattle than those of crossed dairy breeds. Paranhos Da Costa et al. [96] found that male buffaloes feed more from their own mother (2.25 times) and non-filial females (2.4 times), with greater daily weight gain (0.490 ± 0.13 kg/day), than females, who spend more time, on average, suckling from their own mothers (two times) [96,97].

Engelhardt et al. [19] compared the compensation and improved nutrition hypotheses in reindeer (*Rangifer tarandus*). Regarding compensation, they evaluated 25 animals with their calves to quantify whether newborns benefit when having a low birth mass, insufficient maternal milk supply, or delayed growth. The results showed that, although the number of allosuckling events was not influenced by low birth weight, the same animals increased their mass gain while being more involved in allosuckling bouts (0.46% increase from birth to 67 days old); however, no data were found to support the compensation hypothesis. In contrast, in other species, allosuckling has been reported to respond to deficiencies in newborns, such as domestic cattle (*Bos taurus*). In these, the incidence of allosuckling was higher in calves that suckled from alien mothers, which was associated with low birth weight or an insufficient supply of maternal milk (GEE, X2 (1) = 3:73, *p* = 0.05) [94]. In contrast, in 33 fallow deer (*Dama dama*), despite a high incidence of allosuckling (73%), no data were found that could confirm that fawns allosuckle to compensate for their milk requirements not being met [162].

Another example is found in the work of Réale et al. [163], with mouflon (*Ovis gmelini musimon*) during the lambing season. In this study, the authors found that the lambs performed allosuckling in seasons in which the food resources were limited, and had lower growth rates, which is considered to be a constraint in maternal expenditure. In 10 cattle dams, with 20 twin calves from several-bred crosses who showed a high frequency of allosuckling (in 42% of the bouts), the compensation hypothesis was associated with mothers who did not have enough milk for their young [164].

#### 4.2.3. Improved Nutrition

In river buffalo, calves consume the milk of a non-filial female, despite feeding on their mother, perhaps due to nutritional deficiencies that are compensated by allosuckling [64], although a low growth rate of the donors’ calves has also been reported [79]. On the other hand, there are also situations in which allosuckling does not imply any advantage to the calf, as mentioned by Lee [129] in African elephants (*Loxodonta africana*), where allosuckling events represented only 3.7% (of a total of 1865 events) and were performed on nulliparous females. Therefore, they did not provide milk to the alien offspring, and nutritional improvement was absent. The effect that allosuckling exerts on foster mothers was also investigated in a group of meerkats (*Suricatta suricatta*), where allosuckling females lose around 1.43 ± 44.07 g of weight overnight, representing a non-significant change in their weight. Contrarily, non-allonursing mothers had an average weight gain of 20.07 ± 56.14 g, which implies that there is a very low degree of energy stress that does not represent a risk in these species, where the maintenance of an adequate body condition is important for their survival, fertility, and dominance [149].

A greater frequency of allosuckling is also sex-related, as male and female calves, respectively, may perform this behavior occasionally or frequently. On this topic, Drábková et al. [165] studied a group of 28 farmed red deer over two seasons, recording 1730 episodes of suction during 1696 episodes of lactation in 38 fawns born to 23 does. They classified the fawns as frequent or sporadic allosucklers. They observed at least one allosuckling event in 26 of the 38 fawns. Allosucking behavior began during the first week of the fawn’s life. The male fawns performed more allosuckling than females and their duration of allosuckling was longer. The authors concluded that allosuckling fawns have different nursing behaviors, and that recurrent and sporadic allosucklers should be considered when analyzing this behavior.

##### Effects of Sex and Age of Offspring on Allonursing

Another feature observed is that as the offspring’s exclusive alimentation with the mother’s milk decreases with greater age, the frequency of allosuckling also diminishes. Víchová and Bartoš [94] found, in beef cattle, that as the age of the calves increased, allosuckling tended to decrease. Naturally, this tendency towards a reduced intake of the mother’s milk with greater age has also been observed in red deer in the wild, where nursing ends after six to seven months, and on commercial farms, where weaning may occur between 3 and 12 months of life. This act invariably reduces the duration of the mother–infant bond and fosters the offspring’s independence [160]. Hence, allosuckling also decreases as the age of the alien offspring increases, as Drábková et al. [165] reported for farmed red deer (*Cervus elaphus*).

#### 4.2.4. Immunological Function or Benefit

One of the main benefits of allosuckling for the calves is the possibility to obtain a wide variety of antibodies by feeding on different females, to enhance their immune system and resistance to diseases [80]. The dam’s own offspring and an alien newborn could obtain cross-transfer antibodies that are acquired passively by newborns in the group.

When the dam is exposed to pathogenic microorganisms, the mammary gland produces IgA immunoglobulins through plasmatic cells derived from B lymphocytes in the intestine. These immunoglobulins migrate with the plasmatic cells into the milk 2 to 4 weeks before farrowing, then the transfer of IgG and IgM from the blood to the mammary tissue takes place and peaks at parturition. Therefore, premature births, excessively short drying periods, or induced births can produce low amounts of colostrum in immunoglobins, such as IgG. This is important because colostrum is the fluid that, on average, contains the highest concentration of immunoglobulins; it contains 6 g in 100 g, ranging from 2 to 23%, compared to just 0.1% in milk [109]. In foster cows, suckling by alien calves in the first hours after birth is important for colostrum intake [166]. In contrast, wild pigs seem to prevent suckling from non-filial offspring by separating from the group before farrowing and rejoining 10 days after birth [158]. In another aspect, the mother–young binomial (her own or alien) benefits from allonursing because the newborn’s oral cavity contains a variety of microorganisms, many of which could be pathogenic. Contact with the dam’s tissues can cause cell lysis that activates the production of antibodies, which are transported in the bloodstream to the antigens contained on the cell surface of the microorganisms. As a result, ingesting maternal antibodies through the milk of the real or alien mother protects the young from possible infection. The allosuckling infant receives greater protection because it obtains higher antibody levels from the milk of different dams, especially when we consider that the females may be in distinct stages of antibody production for a certain pathogen, and implies genetic advantages in which some females may have specific alleles that allow them to produce distinct or more effective antibodies than those of the biological mother [95]. The immunological advantages not only refer to those destined for the calf, as Roulin [80] has described an endocrine regulation of allosuckling, where the maintenance of high concentrations of prolactin contributes to the immune reinforcement of the mother.

In rodents (*Octodon degus*), the relationship between communal rearing and the transmission of passive immunity, measured in the ratio of white cells and IgG in the mother and newborns, has been studied. However, none had an association between immunocompetence and communal rearing [167]. In the same species, Becker et al. [168] found that communal breeding offers immunological properties, in which feeding on more than one female allows them to obtain antibodies and defense cells, such as IgG and IgA. Although it was shown that mothers can transfer antibodies to offspring and non-offspring during lactation, the data did not show an immunological improvement to support the hypothesis of immunocompetence.

Finally, it is important to highlight that allosuckling may entail disadvantages for the alien offspring, such as a higher probability of suffering lesions by the responses of particularly aggressive alien dams [124], and a high risk of the transmission of pathogens through the milk [64]. Dalto et al. [169] suggest that allosuckling is a risk factor for contracting Johne’s disease or paratuberculosis in buffalo, while milk production can also be affected in the dam-rearing systems of cattle and buffaloes [170].

### 4.3. Altruism in Allomaternal Care?

Although the mentioned hypotheses seek to explain why females engage in allomaternal behaviors, the probability that non-filial mothers accept alien offspring has also been associated with an act of altruism [64]. In these circumstances, the non-filial offspring succeeds in allosuckling without the presence of the biological calf; for example, Brandlová et al. [171] observed five events of allosuckling in camels (*Camelus bactrianus*) and concluded that there is a probability that females recognize the young as a non-filial calf, but tolerate the approach because the benefit is higher or does not represent a considerable cost. These reasons continue to be research elements for further work in all species in which alloparental care predominates.

The effect of stress deserves a final consideration. In several studies [172,173], suckling is associated with significant alterations in the reactivity of the hypothalamic–pituitary–adrenal (HPA) axis during the postpartum period. During lactation, suckling increases both oxytocin and prolactin release, and decreases the plasma levels of ACTH and cortisol, suggesting an inhibitory influence of lactogenic peptides on the HPA axis [174], which could add positive mental states, reducing stress levels during allonursing/allosuckling.

In general, nowadays, different benefits from allonursing/allosuckling have been suggested; for example, individuals can gain experience by caring for other offspring, which can translate into better reproductive performance when they become mothers [100]. However, in the future, it is necessary to evaluate this and other types of possible advantages, and carry out experiments that allow for controlling the effect that variables such as parity, age, and the female’s own experience may have on those individuals. In addition to the above, it is necessary to increase not only the evolutionary approaches, but also to deepen the physiological mechanisms involved in allonursing/allosuckling. Similarly, within livestock production, it is necessary to evaluate the economic costs or productive and reproductive benefits that this type of behavior can bring.

## 5. Conclusions

The phenomena of allonursing and allosuckling are currently being documented in an ever-greater number of domesticated and wild animal species, including both terrestrial and aquatic mammals. Because allonursing forces alien dams to increase their energy expenditures, by allowing alien offspring to feed at their udders, it is a strategy that most commonly develops within social groups where the group members do not change frequently, and the social structure is maintained for long periods. During allonursing, both the alien offspring and the non-filial mothers obtain the following benefits: improved welfare for the former (by improving immunity, satiating hunger, and satisfying behavioral needs, such as sucking, among others) and better maternal performance for the latter (i.e., increasing experience). However, we cannot lose sight of the other side of the coin, since allonursing also has the potential to compromise animal health by transmitting disease-causing microorganisms. Nevertheless, even this negative aspect has a positive side, since increased antibody production and transport through lactation may benefit both the dam and the offspring. Further studies will be required to more precisely determine the specific benefits that allosuckling and allonursing could provide to dams and offspring, both their own and alien.

## Data Availability

Not applicable.
